# Cognitive dysfunction and hippocampal metabolic alterations in patients with postherpetic neuralgia

**DOI:** 10.3389/fneur.2026.1769693

**Published:** 2026-03-04

**Authors:** Xiaokang Ni, Yanrong Yuan, Jun Wang, Huili Liu, Yan Zhang, Yongxing Yan, Jing Han

**Affiliations:** 1 Department of Psychiatry, Hangzhou Third People’s Hospital, Zhejiang, China; 2 Department of Neurology, Hangzhou Third People’s Hospital, Zhejiang, China

**Keywords:** cellular metabolism, chronic pain, cognitive function, hippocampus, magnetic resonance spectroscopy, postherpetic neuralgia

## Abstract

**Objectives:**

Chronic pain is closely related to cognitive function, and pain caused by different etiologies may impair diverse domains of cognition. However, the change of cognitive function and cellular metabolism in hippocampus in postherpetic neuralgia (PHN) patients have received little attention. This study aimed to investigate the cognitive function and cellular metabolism changes in bilateral hippocampus in patients with PHN.

**Methods:**

From July 2021 to December 2024, 41 PHN patients, 48 acute herpes zoster (HZ) patients and 43 controls were enrolled. ^1^H-magnetic resonance spectroscopy (^1^H-MRS) detected bilateral hippocampal metabolism. Cognitive function, pain intensity, depression and anxiety were assessed via Montreal Cognitive Assessment (MoCA), Numeric Rating Scale (NRS), Hamilton Depression Rating Scale (HAMD) and Hamilton Anxiety Rating Scale (HAMA), the correlation between clinical features and the cellular metabolism of bilateral hippocampal was analyzed.

**Results:**

Cognitive impairment occurred in 31.7% (13/41) of PHN patients, whose MoCA scores were significantly lower than HZ and control groups (*p* < 0.05, *p* < 0.01), mainly involving visualspatial executive function, attention and abstraction (all *p* < 0.05). Binary logistic regression analysis found that duration of disease and NRS scores were independent risk factors for cognitive impairment in PHN patients (*p* < 0.05). The Choline/Creatine (Cho/Cr) levels in the bilateral hippocampus of patients in the PHN group were significantly lower than those in the HZ and the control group (*p* < 0.05, *p* < 0.01), and the N-acetylaspartate/Creatine (NAA/Cr) levels in the bilateral hippocampus of patients in the PHN group were significantly lower than those in the control group (*p* < 0.05). The duration of disease and NRS scores of PHN patients were negatively correlated with bilateral hippocampal Cho/Cr levels and MoCA scores (*p* < 0.05, *p* < 0.01), a positive correlation between HAMD/HAMA scores (*p* < 0.01).

**Conclusion:**

PHN patients have a high incidence of cognitive impairment, mainly characterized by reduced visualspatial execution, attention and abstraction abilities, they also exhibit metabolic changes in bilateral hippocampus. Among them, PHN patients with long duration of disease and severe pain have more significant changes. For the management of PHN patients, clinicians should not only pay attention to the patient’s pain symptoms, but also to their cognitive and emotional disorders.

## Introduction

In addition to abnormal pain perception, cognitive impairment associated with chronic pain has garnered significant attention in recent years. Several studies have shown that patients with chronic pain are often accompanied by cognitive decline, including attention, learning and memory, information processing speed, psychomotor ability and executive function, chronic pain of different etiologies may lead to distinct impairments across cognitive domains (1–5). Bell et al. (6) found that persistent pain interference was significantly associated with poorer cognitive performance, cognitive impairment or subjective memory decline in patients, with chronic pain-related cognitive impairment increasing by 21% every 2 years. Smith et al. (7) identified an association between pain and mild cognitive impairment (MCI), suggesting chronic pain may be a risk factor for MCI. Therefore, integrating early identification and intervention for chronic pain-related cognitive abnormalities into pain management holds significant clinical and societal value in helping chronic pain patients reduce their risk of dementia.

Herpes zoster (HZ) is an infectious skin disease caused by the reactivation of the varicella-zoster virus (VZV) latent in the human body, characterized by skin blisters, erythema, and pain. Epidemiological studies (8, 9) indicate that the incidence of herpes zoster is increasing annually. Concurrently, patients with HZ often develop various complications, among which postherpetic neuralgia (PHN) is the most common one with a high incidence rate, and also a prevalent type of chronic pain. Studies have reported that approximately 5–30% of HZ patients will progress to PHN. Zhang et al. (10) reported incidence, recurrence and hospitalization rates for PHN as 12.6% (95% CI:10.1–15.1), 9.7% (95% CI:3.2–16.2) and 6.0/100,000 (95% CI: 2.3–14.2), respectively. Additionally, approximately 30–50% of PHN patients experience persistent pain lasting over 1 year, with some cases persisting for 10 years or longer (11). PHN is readily diagnosed due to its characteristic herpes history, neurotomal distribution of skin lesions, and typical pain or sensory abnormalities within the affected area. However, patients often miss the optimal treatment window or receive inappropriate treatment, which easily leads to refractory or intractable chronic pain, making subsequent treatment extremely challenging and seriously affecting patients’ quality of daily life both psychologically and physically. Although PHN is a common chronic painful disorder with distinct etiology and pathogenesis from other chronic pain conditions, the impact of multiple pain dimensions (such as location, intensity and interference) on cognition also varies (6, 12). Nevertheless, as a modifiable potential risk factor, there are few studies on the relationship between PHN and cognitive impairment currently. Therefore, this study aimed to explore changes in cognitive function and psychological status in PHN patients, and analyze alterations in hippocampal cellular metabolism, so as to provide clues and scientific basis for the prevention strategies of PHN-related cognitive impairment.

## Materials and methods

### Patients and grouping

This retrospective study enrolled 41 patients with PHN and 48 patients with acute HZ admitted to our hospital from July 2021 to December 2024. Meanwhile, 43 healthy individuals during the same period were selected as the control group. The study was approved by the ethics committee of the Hangzhou Third People’s Hospital (No: 2025KA272).

### Inclusion and exclusion criteria

Inclusion criteria of HZ and PHN Patients: The diagnosis of HZ conformed to the criterion of European consensus-based guideline on the management of the herpes zoster (13), with HZ onset within 2 weeks; The diagnosis of PHN met the criteria for chronic pain established by the International Association for the Study of Pain (IASP) (14), with a numerical rating scale (NRS) score exceeding 3 points. All enrolled patients were aged over 18.

Exclusion criteria: (1). Patients with other chronic pain except for PHN; (2). Patients with a history of depression, anxiety, or cognitive impairment; (3). Patients with any impairment of sight, speech, or hearing that would impair the tests for cognition, pain, depression and anxiety; (4). People with severe liver and kidney dysfunction [Child-Pugh Grade B or lower, or an estimated glomerular filtration rate (eGFR) < 30 mL/(min·1.73m^2^)]; (5). Incomplete clinical data records (it required that all analyzed data be complete).

Control group: Healthy volunteers who underwent physical examinations at our hospital during the same period were enrolled. All control individuals met the following criteria: (1) Aged ≥ 18 years; (2) No history of chronic pain, herpes zoster or associated neuralgia; (3) No history of neuropsychiatric disorders such as depression, anxiety or cognitive impairment; (4) No severe sensory/communication disorders that may affect assessment (e.g., obvious visual, auditory or language impairments).

**Figure 1:**
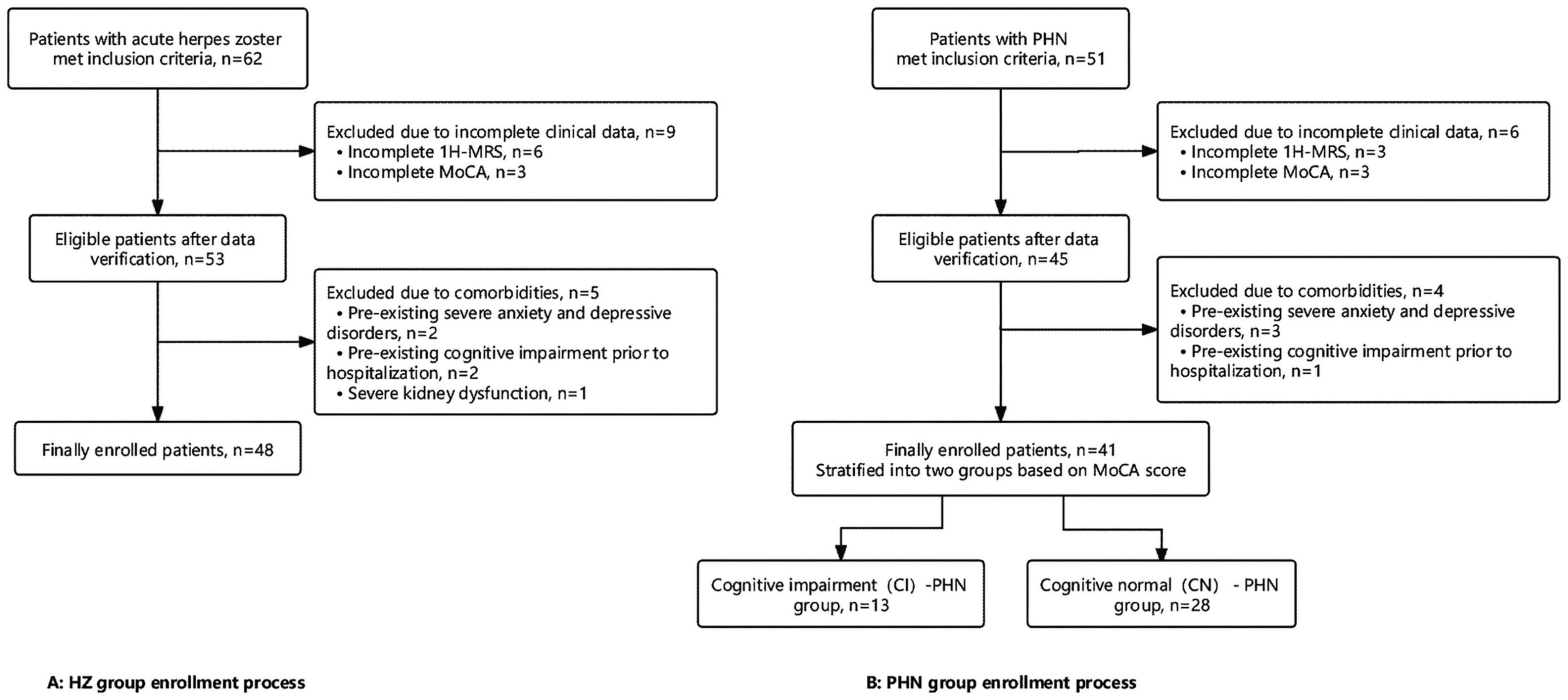
Study flowchart.

A detailed flowchart illustrating the patient screening and enrollment process for this retrospective study is presented in Figure 1.

## Methods

### Collection of patient demographic data

Data including age, gender, location of herpes, years of education and comorbidities of PHN and HZ patients were extracted from the hospital electronic medical record system. Meanwhile, baseline demographic information for the control group was collected.

### Assessment of depression, anxiety, pain intensity, and cognitive function

All patients underwent evaluations for depression, anxiety, pain intensity and cognitive function within 48 h of admission. All assessments were conducted by uniformly trained evaluators.

The Hamilton Depression Rating Scale (HAMD; 17-item version) and Hamilton Anxiety Rating Scale (HAMA) were used to assess the severity of depressive and anxiety disorders. Higher scores indicate more severe depressive or anxiety disorders. A HAMD total score >17 confirmed depressive disorder, while a HAMA total score >13 confirmed anxiety disorder. The NRS was used to evaluate pain intensity, categorized as follows: 0 = no pain, 1–3 = mild pain, 4–6 = moderate pain, and 7–10 = severe pain. Cognitive function was assessed in all enrolled patients using the Chinese version of the Montreal Cognitive Assessment (MoCA). The scale assesses cognitive domains including visualspatial executive function, naming, delayed memory, orientation, attention, verbal fluency and abstraction. The total scores are 30 points, with higher scores indicating better cognitive function (a 1-point adjustment is applied to test results for individuals with ≤12 years of education). A score <26 indicates cognitive impairment.

### ^1^H-magnetic resonance spectroscopy assessment of bilateral hippocampal cellular metabolism

All patients underwent ^1^H-MRS examinations within 48 h of admission (15). Following routine cranial MRI scanning with the turbo spin-echo (TSE) sequence (included T1-weighted imaging (T1WI), T2-weighted imaging (T2WI) and fluid-attenuated inversion recovery (FLAIR)), ^1^H-MRS scanning was performed using the multi-voxel chemical shift imaging (CSI) sequence with point-resolved spectroscopy (PRESS). The scanning parameters were set as follows: echo time (TE) = 135 msec, repetition time (TR) = 1,600 msec, field of view (FOV) = 240 mm × 240 mm, matrix = 18 × 18, and voxel size = 20 mm × 20 mm × 10 mm. Axial T1WI was used for localization, and the bilateral hippocampal level of the temporal lobe at the midbrain level was selected as the region of interest (ROI). Signal spectra were acquired via gradient selection combined with phase encoding. Water suppression was achieved using the chemical shift selective saturation (CHESS) method, and saturation bands were routinely applied in the PRESS sequence to eliminate interference from surrounding tissues, the total spectral acquisition time was approximately 5 min. After the examination, the original ^1^H-MRS images were co-registered with axial T1WI using the built-in spectral analysis and processing software of the MRI scanner. Baseline calibration and metabolite identification were completed automatically, and the ratios of the area under the curve (AUC) of each metabolite were calculated (i.e., NAA/Cr, Cho/Cr, and NAA/Cho).

### Statistical analysis

This study enrolled three groups of participants, including to the acute HZ group, PHN group and healthy control group, one-way analysis of variance (ANOVA) for intergroup comparisons was applied in our statistical analysis. Based on calculations using G*Power 3.1 software, with a preset significance level of *α* = 0.05 and a moderate effect size (*f* = 0.3), the minimum total sample size required to achieve a statistical power of 80% (1-*β* = 0.8) was determined to be 111 cases.

Data processing and statistical analysis were performed using SPSS 25.0 software. Count data were expressed as frequencies and percentages. Intergroup comparisons employed chi-square tests or Fisher’s exact tests. The Kolmogorov–Smirnov test was employed to assess normality of distribution for each dataset. Normally distributed quantitative data were presented as mean ± standard deviation (x ± sd). Comparisons of means across multiple groups were conducted using ANOVA, while pairwise comparisons between two groups were performed using the S-N-K test. Factors demonstrating statistically significant differences in the univariate analysis were selected for multivariate logistic regression analysis. Correlation analysis employed Pearson’s correlation coefficient. *p* < 0.05 was considered statistically significant.

## Results

### Baseline characteristics of the three groups

Based on sample size calculation, this study requires at least 111 participants. To reduce sample bias, we strive to include a more individuals. According to the inclusion and exclusion criteria (Study flowchart Figure 1), this study ultimately included 132 individuals (including 41 PHN patients, 48 patients with HZ and 43 control subjects). Sensitivity power analysis (*α* = 0.05, Power = 0.8) was conducted on the one-way ANOVA test of the actual included patients in this study, the results showed that based on the actual sample size (132 cases), we have the ability to detect the minimum effect size *f* = 0.27, indicating a medium effect size.

41 PHN patients group comprising 26 males and 15 females, with a mean age of 67.6 ± 9.9 years and an educational attainment of 7.5 ± 4.3 years. The HZ group comprised 48 patients (22 males, 26 females) with a mean age of 63.2 ± 13.8 years and mean educational attainment of 8.3 ± 4.4 years. The control group included 43 patients (28 males, 15 females) with a mean age of 66.1 ± 15.0 years and mean educational attainment of 8.2 ± 4.1 years. No statistically significant differences were observed between the three groups in terms of gender, age, years of education and comorbidities (such as hypertension, diabetes mellitus, coronary heart disease, stroke and malignancy; *p* > 0.05; Table 1).

**Table 1:** Baseline characteristics of patients in the 3 groups on admission.

Characteristics	PHN group (*n* = 41)	HZ group (*n* = 48)	Control group (*n* = 43)	F/ x^2^	*p*
Age(mean ± SD)(years)	67.6 ± 9.9	63.2 ± 13.8	66.1 ± 15.0	1.289	0.279
Years of education (years)	7.5 ± 4.3	8.3 ± 4.4	8.2 ± 4.1	0.390	0.678
Gender					
Male (numbers, %)	26(63.4%)	22 (45.8%)	28(65.1%)	4.28	0.118
Female (numbers, %)	15(36.6%)	26 (54.2%)	15(34.9%)		
Comorbidity (cases)					
Hypertension	21	21	NA	0.495	0.482
Diabetes	6	7	NA	0.001	0.995
Coronary heart disease	3	2	NA	0.414	0.520
Stroke	3	4	NA	0.315	0.859
Tumor	3	3	NA	0.040	0.841
Herpes zoster site (numbers)					
Head and face	11	17	NA	0.756	0.385
Neck	3	7	NA	1.171	0.279
Chest and back	16	11	NA	2.715	0.099
Waist	7	12	NA	0.826	0.363
Limb	5	6	NA	0.002	0.965
HAMD	20.0 ± 13.4	11.6 ± 8.0	5.4 ± 3.2	27.040	0.001
HAMA	19.2 ± 11.6	14.6 ± 12.0	5.1 ± 3.0	22.720	0.001
NRS	5.0 ± 1.8	3.1 ± 1.8	NA	4.927	0.001

NA, not applicable.

### Analysis of cognitive function, depression, anxiety and pain intensity among three groups

The MoCA scores of PHN group were 26.3 ± 2.4, 13 out of 41 PHN patients had cognitive impairment (13/41 = 31.7%), In the HZ group with a MoCA scores of 27.7 ± 1.5, 4 patients (4/48 = 8.3%) demonstrated cognitive impairment. The control group showed no cases of cognitive impairment, with a MoCA scores of 27.9 ± 1.3. Significant differences were observed between three groups (*F* = 10.34, *p* < 0.01). Patients in the PHN group demonstrated markedly lower MoCA scores than those in the HZ group and control group (*p* < 0.01), while no significant differences were observed between the HZ and control groups (*p* > 0.05). Further comparisons revealed that, relative to the control group, patients in the PHN group exhibited reduced visualspatial execution function (*p* < 0.05), attention (*p* < 0.01), language (*p* < 0.05) and abstraction (*p* < 0.05). Compared with the HZ group, patients in the PHN group exhibited reduced visualspatial executive function (*p* < 0.05), attention (*p* < 0.01), and abstraction (*p* < 0.05; Table 2).

**Table 2:** Comparison of cognitive function scores between the 3 groups (x ± sd).

Variable	PHN group (*n* = 41)	HZ group (*n* = 48)	Control group (*n* = 43)	F	*p*
MoCA total scores	26.3 ± 2.4	27.7 ± 1.5**	27.9 ± 1.3**	10.340	0.001
Visualspatial executive function	3.7 ± 1.0	4.2 ± 1.0*	4.2 ± 0.9*	3.427	0.036
Naming	2.8 ± 0.4	2.9 ± 0.3	2.8 ± 0.4	0.848	0.431
Attention	4.7 ± 0.8	5.2 ± 0.8**	5.4 ± 0.8**	7.608	0.001
Language	2.8 ± 0.4	2.9 ± 0.2	3.0 ± 0.0*	3.797	0.025
Abstraction	1.8 ± 0.4	2.0 ± 0.2*	2.0 ± 0.2*	4.063	0.020
Delayed recall	4.3 ± 0.7	4.5 ± 0.6	4.6 ± 0.5	1.639	0.198
Orientation	5.8 ± 0.4	5.9 ± 0.4	5.8 ± 0.4	0.053	0.948

Compared with PHN group: *p < 0.05; **p < 0.01.

Depressive disorder was present in 36.6% (15/41) of PHN group, with a HAMD scores of 20.0 ± 13.4, significantly higher than both the HZ (5/48 = 10.4%) and the control groups (11.6 ± 8.0; 5.4 ± 3.2; *p* < 0.01). Anxiety disorders were present in 46.3% (19/41) of patients in the PHN group, with a HAMA scores of 19.2 ± 11.6, significantly higher than both the HZ (9/48 = 18.8%) and the control groups (14.6 ± 12.0; 5.1 ± 3.0; *p* < 0.01). Furthermore, HAMD and HAMA scores in the HZ group were markedly higher than in the control group (*p* < 0.01), as shown in Table 1.

Patients in the PHN group exhibited significantly higher NRS scores than those in the HZ group (5.0 ± 1.8 vs. 3.1 ± 1.8), with a statistically significant difference (*p* < 0.01; Table 1).

### Independent risk factor analysis of PHN concurrent cognitive impairment

Forty-one patients with PHN were categorized into a cognitive impairment (CI)-PHN group (13 cases) and a cognitive normal (CN)-PHN group (28 cases) based on the presence of cognitive impairment. Intergroup comparisons revealed that CI-PHN patients exhibited significantly higher duration of disease, HAMD, HAMA and NRS scores compared to the CN-PHN group (all *p* < 0.05), while showing significantly lower years of education (*p* < 0.01; Table 3).

**Table 3:** Baseline characteristics of patients in the CI-PHN and CN-PHN groups on admission.

Characteristics	CI-PHN group (n = 13)	CN-PHN group (*n* = 28)	t/ x^2^	*p*
Age(mean ± SD)(years)	71.9 ± 8.2	65.6 ± 10.2	1.961	0.057
Years of education (years)	4.8 ± 4.2	8.8 ± 3.9	2.972	0.005
Gender				
Male (numbers, %)	8(61.5%)	18(64.3%)	0.029	0.865
Female (numbers, %)	5(38.5%)	10 (35.7%)		
Comorbidity (cases)				
Hypertension	7	14	2.455	0.117
Diabetes	1	4	0.009	0.926
Coronary heart disease	2	1	1.827	0.177
Stroke	1	3	0.004	0.949
Tumor	1	1	1.827	0.177
Duration of disease (days)	154.4 ± 33.3	125.9 ± 14.8	3.820	0.001
HAMD	26.3 ± 15.9	17.0 ± 11.2	2.159	0.037
HAMA	25.5 ± 13.8	16.3 ± 9.4	2.501	0.017
NRS	6.3 ± 1.8	4.3 ± 1.4	3.920	0.001
MoCA	23.3 ± 1.2	27.6 ± 1.2	10.670	0.001

CI-PHN, cognitive impairment-postherpetic neuralgia; CN-PHN, cognitive normal-postherpetic neuralgia.

Taking the presence of cognitive impairment as the dependent variable, we conducted a multivariate binary logistic regression analysis using age, gender and the statistically significant factors from univariate analysis (duration of disease, HAMD/HAMA/NRS scores, years of education) as independent variables. It revealed that duration of disease and NRS scores were independent risk factors for cognitive impairment in PHN patients (*p* < 0.05). After adjusting for confounding factors including age, HAMD, HAMA, years of education and comorbidities, duration of disease and NRS scores remained independent risk factors for cognitive impairment in PHN patients (*p* < 0.05; Table 4).

**Table 4:** Binary logistic regression analysis of PHN patients concurrent cognitive impairment and various factors.

Variable	*β* value	SE value	Wald	OR value	95% CI	*p* value
Age	0.116	0.089	1.714	1.123	0.944–1.337	0.190
Gender	0.098	0.137	0.508	1.103	0.843–1.443	0.476
Years of education	−0.101	0.182	0.307	0.904	0.633–1.291	0.579
Duration of disease (days)	0.177	0.088	4.037	1.193	1.004–1.418	0.041
HAMD	0.267	0.157	2.916	1.765	1.563–2.040	0.088
HAMA	0.081	0.092	0.769	1.922	1.770–2.105	0.381
NRS	1.217	0.597	4.148	3.376	1.047–10.883	0.042

Nagelkerke R2 = 0.692; Cox & Snell R2 = 0.494.

### Bilateral hippocampal cellular metabolic alterations

**Figure 2:**
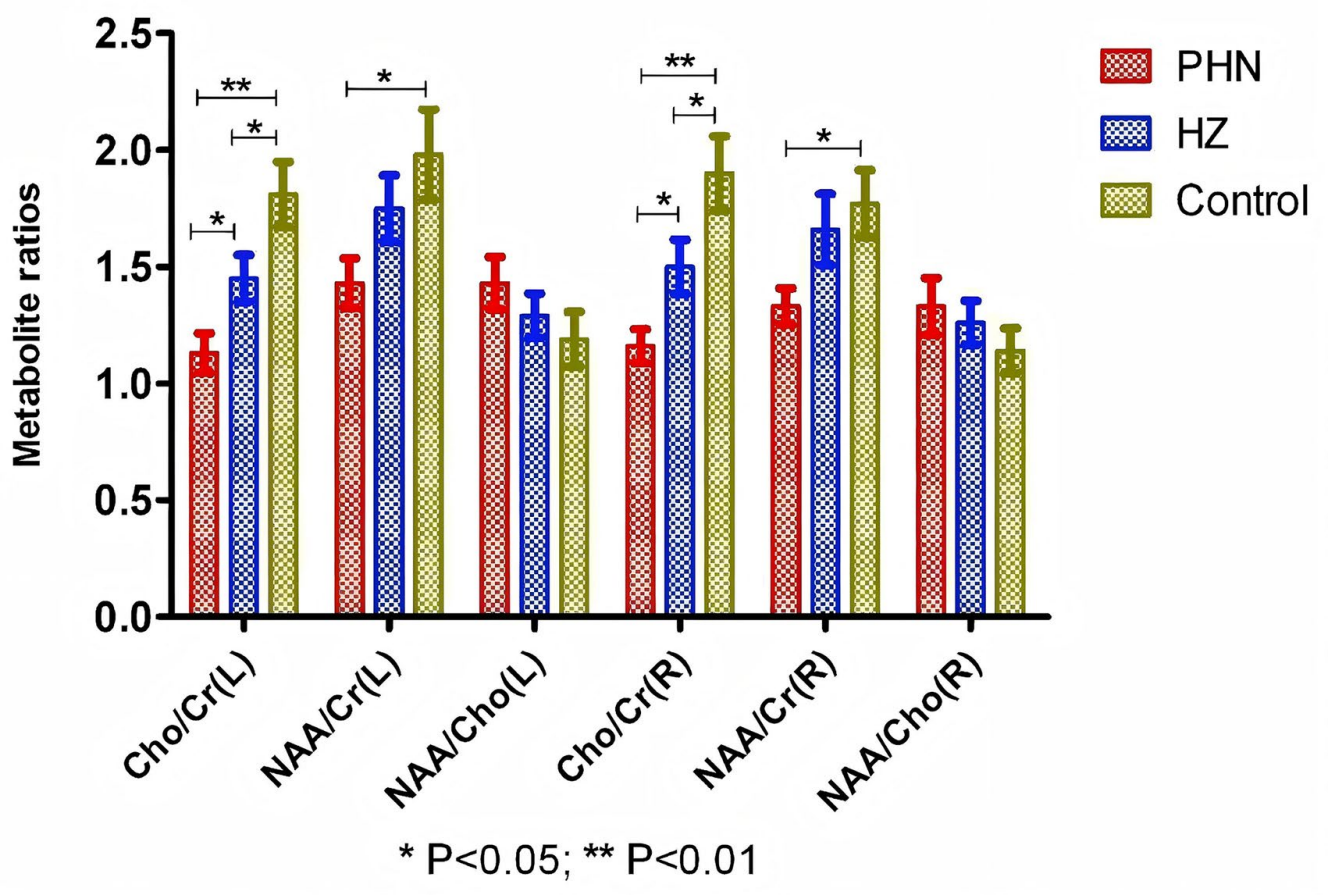
Bilateral hippocampal cellular metabolic alterations between the 3 groups.

^1^H-MRS examination revealed significantly lower Cho/Cr levels in the left and right hippocampal regions of PHN patients compared to HZ patients and controls. (1.13 ± 0.54, 1.16 ± 0.46 vs. 1.45 ± 0.69, 1.50 ± 0.80 vs. 1.81 ± 0.91, 1.90 ± 1.04), differences were statistically significant (all *p* < 0.05). Furthermore, NAA/Cr levels in the left and right hippocampal regions of PHN patients were markedly lower than those in the control group (1.43 ± 0.68, 1.98 ± 1.27; vs. 1.33 ± 0.50, 1.77 ± 0.94; all *p* < 0.05). Concurrently, the left and right hippocampal Cho/Cr levels in the HZ group were lower than those in the control group (*p* < 0.05). However, comparisons of hippocampal NAA/Cho levels between groups showed no statistically significant differences (*p* > 0.05; Figure 2).

### Correlation analysis between clinical characteristics of PHN patients and hippocampal cellular metabolism

Duration of disease, NRS scores of PHN patients showed negative correlations with MoCA scores and left and right hippocampal Cho/Cr ratios (*r* = −0.581, *p* = 0.001; *r* = −0.341, *p* = 0.029; *r* = −0.309, *p* = 0.049; *r* = −0.531, *p* = 0.001; *r* = −0.452, *p* = 0.003; *r* = −0.478, *p* = 0.002), while showing positive correlations with HAMD and HAMA scores (*r* = 0.862, *p* = 0.001; *r* = 0.622, *p* = 0.001; *r* = 0.472, *p* = 0.002; *r* = 0.680, *p* = 0.001). PHN patients’ MoCA scores were positively correlated with bilateral hippocampal Cho/Cr ratios (*r* = 0.458, *p* = 0.003; *r* = 0.331, *p* = 0.034; Table 5).

**Table 5:** Correlation analysis between clinical characteristics and hippocampal cellular metabolism levels in PHN patients.

Variable	Duration of disease		NRS		HAMD		HAMA		Moca		Left of Cho/Cr	
	*r*	*p*	*r*	*p*	*r*	*p*	*r*	*p*	*r*	*p*	*r*	*p*
NRS	0.513	0.001										
HAMD	0.862	0.001	0.472	0.002								
HAMA	0.622	0.001	0.680	0.001	0.597	0.001						
MoCA	−0.581	0.001	−0.531	0.001	−0.422	0.006	−0.499	0.001				
Left of Cho/Cr	−0.341	0.029	−0.452	0.003	−0.390	0.012	−0.287	0.069	0.458	0.003		
Right of Cho/Cr	−0.309	0.049	−0.478	0.002	−0.276	0.080	−0.194	0.224	0.331	0.034	0.451	0.003

## Discussion

Accumulating evidence indicates that the incidence of HZ and PHN exhibits an annual upward trend (8, 9, 16), with the associated pain linked to multiple adverse health outcomes including falls, disability, anxiety, depression and cognitive decline (17–19). This study evaluated cognitive function, affective disorders and hippocampal cellular metabolic levels in patients with PHN, and compared with those in patients with acute HZ and healthy controls. Our study identified three main findings. First, the incidence of cognitive impairment was high in PHN patients, which was predominantly characterized by impairments in visualspatial executive function, attention and abstraction. Second, PHN patients also had a high incidence of depressive and anxiety disorders, with 36.6 and 46.3%, respectively, which was consistent with previous reports (20). Third, the cellular metabolic ratios (Cho/Cr, NAA/Cr) in the bilateral hippocampus of PHN patients were significantly decreased. Notably, longer duration of disease and severe pain intensity were independent risk factors for cognitive impairment in PHN patients, and were significantly correlated with reduced hippocampal Cho/Cr levels and cognitive function decline. These findings establish a direct link between PHN-related clinical manifestations and hippocampal cellular metabolic alterations, providing new evidence for understanding the widespread impact of PHN as a chronic neuropathic pain on the central nervous system. A cross-sectional study by Pickering et al. (21) revealed that vigilance, decision making and semantic memory were significantly impaired in patients with PHN, when compared with healthy controls matched by gender and age. This study explored several domains of cognition in patients suffering from PHN, with onset exceeding 3 months, in accordance with the IASP criteria. Results indicated that 31.7% of PHN patients exhibited cognitive impairment. This suggests clinicians should focus on pain symptoms in PHN patients but also pay attention to non-somatic symptoms such as cognitive function, depression and anxiety. Meanwhile, this study found that cognitive impairment in PHN patients is mainly characterized by decreased visualspatial executive function, attention and abstraction. Without a chronic pain comparator group, so it is difficult to determine whether the findings represent PHN-specific vulnerability or a broader pain-related phenomenon. Further controlled studies are needed to clarify this phenomenon in the future.

Numerous cross-sectional studies indicate that chronic pain is closely associated with impairments across multiple cognitive domains, including memory, attention, information processing speed and executive function (5). However, longitudinal research findings regarding the impact of pain duration on cognition remain inconsistent. Some studies indicate that persistent pain accelerates cognitive impairment and increases dementia risk (22), while others find no significant association (4). Furthermore, the cognitive effects vary across different dimensions of pain (e.g., location, intensity, interference) (6, 12), with current research yet to reach definitive conclusions. The present study observed that patients with PHN had not only a high prevalence of cognitive impairment but also a remarkably high incidence of depressive and anxiety disorders, with the rates reaching 36.6 and 46.3%, respectively. This finding indicates a high comorbidity of cognitive and affective symptoms in these patients (23). Accumulating evidence suggests that neuropathic pain leads to the progressive development of depressive symptoms in a time-dependent manner, which is closely associated with profound hippocampal changes such as compromised neurogenesis (24). However, further multivariate analysis revealed that pain intensity (NRS scores) and duration of disease remained independent predictors of cognitive impairment even after adjusting for multiple confounding factors, including depressive and anxiety scores. This demonstrates that PHN-associated chronic pain poses a direct and independent risk to cognitive function, rather than being merely a secondary effect of concomitant emotional disorders, the conclusion further supported by our neuroimaging findings. Consistent with prior evidence, mice with neuropathic pain display substantially reduced hippocampal neurogenesis, which may serve as a key structural basis for pain-related cognitive dysfunction (25). Patients with PHN exhibited a significant reduction in the levels of cellular metabolites in the bilateral hippocampal regions, and the severity of these metabolic abnormalities was significantly negatively correlated with pain intensity and duration of disease, whereas only a weak correlation was observed between such abnormalities and depressive/anxiety scores. These results provide biological evidence that chronic pain directly or indirectly induces hippocampal dysfunction. Of course, affective disorders and chronic pain may exert a synergistic effect, jointly amplifying or accelerating the decline in cognitive function (26). Therefore, for cognitive impairment in PHN patients, we speculate that chronic pain, affective disorders (depression/anxiety) and cognitive impairment exist within an intricate, mutually exacerbating network. Specifically, chronic pain itself acts as the core pathophysiological driver of cognitive impairment (27), while the highly prevalent depressive and anxiety disorders which common psychological sequelae of chronic pain may further exacerbate cognitive deficits by exacerbating the loss of neuroblasts and reducing survival of new mature neurons in hippocampal granular layers (26). Therefore, further research is warranted to elucidate the causal relationships among these three factors and to explore comprehensive intervention strategies targeting this complex comorbid state in PHN patients.

The hippocampus is essential for encoding/early consolidation and recent memory (28). Recent research indicates an association between pain and the hippocampus. For instance, pain-related fear activates brain areas including the hippocampus, inferior frontal gyrus, middle frontal gyrus, postcentral gyrus, middle temporal gyrus, paroccipital sulcus and striatum (29). Reactivation of the hippocampus, particularly its anterior region is closely associated with pain memory. The intensity of pain evoked by such memories can alter hippocampal function, with the strength of hippocampal reactivation positively correlating with avoidance behavior associated with aversive pain-related scenarios (30). Many studies have suggested that in humans and rodents, in addition to the nucleus accumbens (NAc) is involved in modulating pain, neuropathic pain and inflammatory induce neurogenesis in the NAc (31–33), adult hippocampal neurogenesis was also found in both neuropathic pain and inflammatory persistent states (34–36). Chronic pain influences hippocampal structure, functional connectivity, electrophysiological properties, neural circuits and neural activity (37). Corresponding hippocampal alterations are also key factors in comorbidities such as pain hypersensitivity, negative emotions and cognitive impairment observed during chronic pain (38, 39). Although chronic pain patients exhibit hippocampal alterations, the specific hippocampal abnormalities in PHN patients remain unclear. As a non-invasive imaging technique revealing *in vivo* tissue metabolism, MRS can assess tissue changes from a metabolic perspective prior to morphological alterations. It is currently understood that NAA is primarily located within neuronal cell bodies and synapses, serving as a sensitive indicator of neuronal damage. Its concentration directly reflects neuronal density; following neuronal injury, NAA levels decrease. Cr serves as a marker for energy metabolism in brain tissue cells. Cho constitutes a component of cell membranes, with its concentration determining membrane function. Therefore, this study employed MRS to investigate cellular metabolic changes in the bilateral hippocampal regions of PHN patients. Results revealed significantly lower Cho/Cr levels in both left and right hippocampal regions of PHN patients compared to both the HZ and control groups. NAA/Cr levels in both left and right hippocampal regions of PHN patients were markedly lower than those in the control group whereas there was no difference in bilateral NAA/Cr between PHN and HZ groups. These findings may reflect a “selective effect” of chronic pain on hippocampal metabolism, suggesting that chronic pain preferentially disrupts membrane metabolism-related signaling pathways. Whether a longer disease duration in PHN patients leads to hippocampal neuronal death and more pronounced abnormalities in bilateral NAA/Cr ratios warrants further investigation in future studies. These findings reveal distinct metabolic alterations within the bilateral hippocampus in patients with PHN. Further analysis of the correlation between clinical manifestations and hippocampal cellular metabolism revealed that duration of disease and NRS scores in PHN patients were negatively correlated with left and right-sided Cho/Cr ratios. This further elucidates the role of the hippocampus in chronic pain. PHN patients exhibit not only cognitive impairment but also disrupted hippocampal cellular metabolism. The severity of pain and duration of disease correlate with more pronounced abnormalities in PHN patients. These findings provide scientific rationale for developing novel therapeutic strategies and offer new perspectives for treating chronic pain and its associated cognitive impairment complications.

This study also has certain limitations. First, being a single-center, retrospective study with a small sample size, and the number of enrolled PHN patients was limited which directly leads to a significant reduction in the reliability of subgroup analyses. Owing to the small sample size, the results may be subject to bias, which consequently limits the generalizability of our conclusions. Second, it did not dynamically observe changes in cognitive function or MRS in PHN patients. Comparing the dynamic relationship between these would provide more valuable reference for clinicians; the third, previous studies have found abnormalities in hippocampal cellular metabolism in patients with depression and anxiety. This study identified that some PHN patients with cognitive impairment also exhibited depressive and anxiety disorders. Due to the small sample size, these patients were not excluded, potentially influencing the results. Nevertheless, the co-occurrence of depression, anxiety and cognitive impairment in PHN patients in the clinical real-world renders this study more clinically relevant. Naturally, future large-scale, multi-center prospective clinical studies are required to further validate these findings.

## Conclusion

In summary, a close association exists between pain and cognitive impairment. As a common chronic painful condition, PHN carries a high incidence of co-occurring cognitive impairment, primarily manifesting as a cognitive phenotype characterized by reduced visualspatial executive function, attention and abstraction. It is accompanied by cellular metabolic alterations in the bilateral hippocampal regions, with more pronounced changes observed in patients with longer duration of disease and more severe pain. Therefore, in managing PHN patients, clinicians should not only address pain symptoms but also monitor cognitive function and emotional disturbances such as depression and anxiety, thereby enhancing patients’ quality of life.

## Data Availability

The raw data supporting the conclusions of this article will be made available by the authors, without undue reservation.
